# Physics-Guided Dual-Branch Fusion Model for High-Resolution Range Profile Target Recognition

**DOI:** 10.3390/s26113608

**Published:** 2026-06-05

**Authors:** Ziheng Xia, Mengdie Wu, Feng Xiao, Hongwei Liu

**Affiliations:** 1 School of Defence Science and Technology, Xi’an Technological University, Xi’an 710021, China; xffriends@xatu.edu.cn; 2 National Key Laboratory of Radar Signal Processing, Xidian University, Xi’an 710071, China; 3 School of Computer Science and Engineering, Xi’an Technological University, Xi’an 710021, China; wumengdie@st.xatu.edu.cn

**Keywords:** high-resolution range profile (HRRP), radar automatic target recognition (RATR), dual-branch fusion, physics-guided learning, sparse peak parameter modeling

## Abstract

High-resolution range profile (HRRP) target recognition has advanced with deep learning, yet most existing methods rely primarily on data-driven feature extraction and still face challenges in physical interpretability and noise robustness. To address these issues, this paper proposes a physics-guided dual-branch fusion (PGDBF) model. It consists of two parallel branches: a data-driven branch extracts discriminative features from raw HRRPs, while a physics-guided branch estimates sparse peak parameters (position and intensity) and reconstructs the signal envelope under sparsity constraints. Cross-attention adaptively fuses the two branches. Experiments on measured ten-class aircraft HRRP data show that PGDBF achieves higher accuracy and improved robustness under additive Gaussian noise in the evaluated fixed-route scenario. Visualizations confirm that the estimated peaks align with dominant HRRP energy, linking model variables to physically meaningful peak locations and intensities. These results suggest that integrating explicit peak parameter estimation with data-driven learning is a promising direction for improving HRRP recognition robustness and interpretability under low-SNR conditions.

## 1. Introduction

Radar automatic target recognition (RATR) plays a critical role in modern defense and security systems, enabling the identification of targets based on radar echoes. Among various radar signatures, the high-resolution range profile (HRRP) has garnered significant attention because of its practicality and rich information content. HRRP represents the distribution of radar scattering energy along the radar line of sight, capturing important structural characteristics of the target such as size, geometry, and dominant scattering responses [1,2]. Unlike synthetic aperture radar (SAR) or inverse SAR, which require complex imaging processes and extensive data acquisition, HRRP offers a computationally efficient and storage-friendly alternative while retaining essential target-specific features. This advantage makes HRRP particularly suitable for real-time recognition of aerial and space targets.

The evolution of HRRP-based recognition methods has progressed through three key stages: statistical modeling [3,4,5,6], traditional machine learning [7,8,9], and deep learning [10,11,12,13,14]. In the statistical modeling stage, methods such as Gaussian mixture models (GMM) and factor analysis [15] were adopted to characterize HRRP distributions. Traditional machine learning relies on handcrafted features, including peak distribution, centroid and entropy, combined with classic classifiers such as support vector machines (SVM). In recent years, deep learning has dominated this research field, where convolutional neural networks (CNNs) [10,16,17] and Transformer architectures [18,19,20,21] have achieved promising performance on measured HRRP datasets. Nevertheless, existing deep learning methods still suffer from insufficient physical interpretability and poor noise robustness. HRRP signals exhibit sparse dominant peak responses that are highly correlated with target scattering energy distribution, and such peak structures carry crucial discriminative information for classifying targets with similar overall profiles. Most data-driven methods simply treat HRRP as a generic one-dimensional sequence and learn abstract latent features through implicit extraction. Although favorable accuracy can be obtained, it is difficult to guarantee that the learned embeddings preserve physically meaningful peak structures, leading to limited interpretability and high sensitivity to noise perturbations.

Recent studies have introduced physical priors and scattering constraints into deep networks through attention enhancement, subspace projection and feature regularization [13,22,23,24], which improves feature robustness to a certain extent. However, these methods tend to implicitly embed physical information into feature extraction, resulting in learned representations that lack explicit physical correspondence. Dominant peaks in HRRP contain positional and intensity information inherently linked to target scattering characteristics. Explicit modeling of such peak parameters helps retain physically meaningful structural information and enhances interpretability. Unlike implicit feature learning, explicit peak representation directly preserves the position and intensity of dominant scattering components and can be effectively regularized by reconstruction consistency. Nevertheless, such explicit structured peak parameter representations are still less explored in current deep HRRP recognition frameworks.

Based on the above analysis, this work focuses on three key research problems: how to introduce explicit structured peak parameters into deep HRRP recognition frameworks, how to constrain peak parameters to maintain structural integrity and suppress noise, and how to fuse physics-inspired priors with data-driven features. To address these limitations, this paper proposes a Physics-Guided Dual-Branch Fusion Network (PGDBF). The framework explicitly estimates sparse peak parameters from HRRP signals and integrates them with data-driven representations via cross-attention fusion. Reconstruction consistency and sparsity constraints are further imposed to regularize peak parameters, enabling the network to focus on dominant scattering structures and suppress noise interference, thus improving recognition robustness under low-SNR conditions.

The main contributions of this work are as follows:

- An explicit sparse peak parameterization framework is proposed. Unlike existing implicit physics-inspired methods that embed physical priors as abstract regularizations, our method directly estimates peak positions and intensities from HRRPs, producing an interpretable structural representation aligned with dominant HRRP energy. This addresses the lack of physical interpretability in conventional deep models.
- Reconstruction consistency and sparsity constraints are introduced to regularize the peak parameters, jointly suppressing noise-sensitive responses and preserving structural integrity, thereby improving robustness under low-SNR conditions.
- A dual-branch cross-attention fusion mechanism is designed to adaptively integrate explicit peak representations with deep discriminative features, achieving complementary advantages.

Extensive experiments on a measured ten-class aircraft HRRP dataset demonstrate that PGDBF achieves higher overall accuracy than purely data-driven baselines and improves discriminability for structurally similar targets. Under different SNR levels, the proposed method maintains stable performance and outperforms existing competitors, with more remarkable superiority in low-SNR scenarios. Ablation studies verify the effectiveness of dual-branch collaboration and the reconstruction module. Quantitative reconstruction error analysis and t-SNE visualization are also provided to explore the representation characteristics and framework’s effectiveness.

## 2. Related Works

### 2.1. Statistical Models and Traditional Machine Learning Methods

#### 2.1.1. Statistical Models

Early studies established Gaussian-based statistical models as baseline approaches for HRRP recognition [3]. To address the non-Gaussian and multi-modal characteristics of measured HRRP data, Du et al. [4] proposed a two-distribution compounded statistical model. Subsequent works extended statistical modeling: factor analysis (FA, ref. [15]) captures correlations among range cells, local factor analysis (LFA, ref. [2]) models the non-Gaussianity of HRRP data, and multi-task hidden Markov models [25] capture sequential dependencies. Variational autoencoders (VAEs) and their discriminative extensions [26,27,28] have also been introduced. While these models offer some statistical interpretability, they rely on assumptions such as linearity or low-dimensional latent spaces and struggle to capture highly nonlinear HRRP structures.

#### 2.1.2. Traditional Machine Learning

Traditional machine learning approaches follow a feature extraction-then-classification pipeline. Early efforts [29] extracted handcrafted range profile features with simple matching-score decisions. Du et al. [30] used higher-order spectra features with Euclidean distance classification. Subsequent work introduced spectral-domain features [31], translation-invariant features [32], and frame-level features [33]. Subspace learning techniques such as multi-scale sparse preserving projection [8] and discriminant geometry co-learning [9] have also been explored. A common limitation of these methods is that performance is bounded by the quality of handcrafted features, and they cannot learn hierarchical representations automatically.

### 2.2. Data-Driven Deep Learning Approaches

Early deep learning studies demonstrated the feasibility of HRRP classification using CNNs [16,17]. However, standard CNNs struggle with long-term sequential dependencies under varying aspect angles. To address this, hybrid architectures have been proposed, including the target-aware recurrent attentional network (TARAN, ref. [34]) and the stacked CNN-Bi-RNN with attention mechanism (SCRAM, ref. [35]). Multi-scale networks [36] and group-fusion CNNs [37] have been developed for efficiency. Transformer-based methods [18,19,20,21] have also been introduced to capture global dependencies. Recently, Zhang et al. [19] developed a patch-wise autoencoder based on transformer (PwAET). This model splits HRRP into overlapping patches and leverages multi-head self-attention to capture inter-patch correlations, while a decoder reconstructs the original HRRP for enhanced noise robustness. To handle limited labeled data, strategies such as discriminative autoencoders [38], GAN-based augmentation [39], and meta-learning [40] have been investigated. Despite these advances, these methods treat HRRP primarily as a generic 1D signal without explicit modeling of sparse peak distributions.

### 2.3. Physics-Inspired Deep Learning Methods

Recent studies have attempted to incorporate scattering-related priors into deep networks using various strategies. Liu et al. [22] parameterizes a learnable scattering center dictionary, effectively embedding structural priors into the network. Liu et al. [13] designs a customized loss function to enforce aspect-angle consistency, improving generalization across views. Chen et al. [12] introduces variational parameterized convolution to handle azimuth side information, focusing on robustness under imperfect orientation knowledge. Gao et al. [23] aligns attention maps with estimated scattering regions, providing coarse spatial correspondence. Liu et al. [24] unrolls the GoDec algorithm for noise-robust decomposition, producing signal-level components rather than structured peak parameters. Chen et al. [41] integrates discrete wavelet transform and signal subspace projection with SNR prior into a denoising-recognition cascade, achieving noise-robust HRRP recognition. However, similar to the above approaches, its internal representations remain in the signal domain and lack explicit, physically interpretable peak parameters. Though prior efforts have embedded physical priors into networks as inductive biases using multiple strategies, including dictionary learning, loss regularization, attention alignment, algorithm unrolling and transform-domain projection, most of them decouple physical constraints and data-driven optimization. As a result, the latent representations stay abstract and cannot be directly mapped to physically interpretable features.

In contrast, the proposed method aims to bridge this gap by explicitly estimating a sparse set of peak parameters (positions and intensities) as part of the feature representation. Instead of merely regularizing the network, we decompose the recognition process into two synergistic pathways:

- Physics-Guided Parameterization: A dedicated branch estimates sparse peak parameters that directly correspond to dominant HRRP energy distributions. A reconstruction constraint ensures these parameters remain consistent with the measured HRRP envelope.
- Data-Driven Enhancement: A cross-attention fusion module dynamically integrates these explicit parameters with abstract data-driven features, allowing the network to leverage the interpretability of the peak-based representation while retaining the discriminative power of deep learning.

This dual-path mechanism provides a unified framework where physical priors are not just constraints but are transformed into explicit features used for decision making.

## 3. Methodology

**Figure 1:**
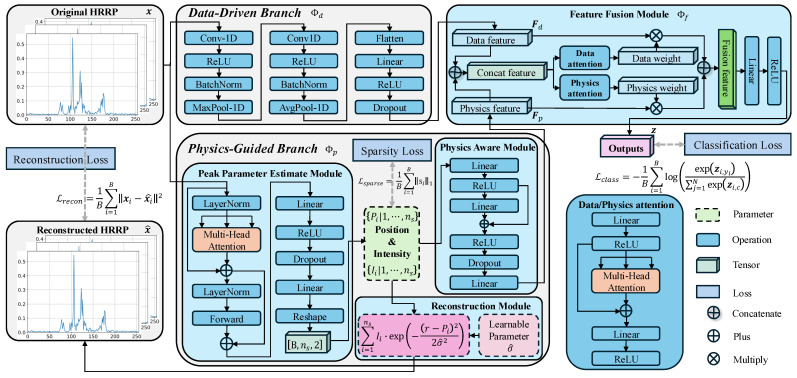
Framework of the proposed PGDBF model.

This section provides a comprehensive description of the proposed method. The overall architecture, as illustrated in Figure 1, is designed to integrate data-driven learning with scattering-inspired constraints in an end-to-end framework. The model consists of two main branches: a data-driven branch that extracts discriminative features from the original HRRP, and a physics-guided branch that estimates sparse peak parameters associated with dominant HRRP energy distributions. These two branches are fused adaptively, and the entire network is optimized under a joint framework with classification, reconstruction, and sparsity regularization objectives.

### 3.1. Overview

The proposed method takes the original HRRP as input, processing it through two parallel pathways. The data-driven branch employs a standard stack of 1D convolutional and linear layers to learn hierarchical representations directly from the data. Simultaneously, the physics-guided branch estimates a set of sparse peak parameters (positions and intensities) from the HRRP and uses these parameters to reconstruct the dominant peak envelope of the input HRRP. A feature fusion module utilizes an attention mechanism to dynamically combine the features from both branches. The model is trained with a multi-loss function that includes a classification loss, a reconstruction loss to enforce consistency with the HRRP peak envelope, and a sparsity loss to promote parsimonious representations.

### 3.2. Data-Driven Branch

This branch functions as a feature extractor, transforming the input HRRP into a high-dimensional feature vector. The input is the original HRRP, denoted as $x\in R^{L}$, where *L* is the length of the range profile. The branch consists of a series of 1D convolutional blocks. Each block typically includes a Conv-1D layer, a ReLU activation function, batch normalization (BatchNorm), and a 1D Max-Pooling layer (MaxPool-1D) for down-sampling. The convolutional stages are followed by fully-connected (Linear) layers with ReLU activation and Dropout for regularization. The final output of this branch is a data feature vector $F_{d}\in R^{d}$.

### 3.3. Physics-Guided Branch

The physics-guided branch approximates the dominant HRRP energy distribution using a sparse set of peak components, which differs from conventional data-driven structures by explicitly introducing peak-based constraints into feature learning.

Peak Parameter Estimation Module: This module estimates a set of sparse peak parameters from the input HRRP using a transformer-based architecture with multi-head attention and residual connections. Unlike convolutional operations with fixed local receptive fields, self-attention mechanisms enable adaptive modeling of long-range dependencies between separated dominant peaks in HRRP sequences. This property is beneficial for HRRP analysis because dominant peaks may exhibit nonlocal interactions and varying spatial separations under different target aspects. Given an input HRRP of size [B,1,L] (where *B* is the batch size and *L* is the range length), the module outputs a tensor of size $\left[B,n_{s},2\right]$. Here, $n_{s}$ is a hyperparameter denoting the number of peak parameters to estimate. The value 2 corresponds to two quantities per peak: the position $\left\{P_{i}\right\}$ and intensity $\left\{I_{i}\right\}$ for $i=1,\ldots ,n_{s}$. To ensure physically meaningful parameter estimation, the position output is passed through a sigmoid activation function, constraining each estimated peak position to the normalized range interval [0,1]. The intensity output is processed using a Softplus activation followed by clipping, ensuring positive and numerically stable intensity estimates. Therefore, all peak parameters remain within physically valid bounds throughout training. Notably, the maximum number of range bins that can be resolved within a target of typical length is limited by radar range resolution, which can be estimated as L/(c/(2BW)), where *L* is the target length, *c* is the speed of light, and BW is the radar bandwidth. In our implementation, we set $n_{s}=32$ for all target categories, which is smaller than the theoretical upper bound determined by the corresponding target size and radar resolution. Sparsity regularization further encourages the model to retain only the dominant peak components.

Physics-Aware Module: The estimated peak parameters $\left\{P_{i},I_{i}\right\}$ are not directly suitable for classification. A physics-aware module, consisting of linear layers, ReLU activations, and a residual connection, transforms these parameters into a physics-guided feature vector $F_{p}\in R^{p}$. This transformation allows the sparse peak parameters to be represented in a feature space compatible with the data-driven branch while preserving coarse peak distribution characteristics.

Reconstruction Module: The reconstruction module serves two purposes: (i) it provides a differentiable mechanism to regularize the estimated peak parameters, and (ii) it enables the model to learn representations that are consistent with the HRRP peak envelope.

The reconstruction design is inspired by the geometric theory of diffraction (GTD). For a wideband radar system, the frequency-domain HRRP can be modeled as the coherent superposition of responses from multiple scattering centers:

(1) $$E\left(f\right)=\sum_{i=1}^{n_{s}}I_{i}{\left(j\frac{f}{f_{0}}\right)}^{\alpha_{i}}\exp\left(-j\frac{4\pi fr_{i}}{c}\right),$$

where $r_{i}$ is the distance to the *i*-th scattering center, $I_{i}$ is the intensity, $\alpha_{i}$ is the GTD exponent corresponding to different scattering types (e.g., specular reflection, edge diffraction), and *c* is the speed of light. The range-domain HRRP h(r) is obtained via inverse Fourier transform:

(2) $$h\left(r\right)=F^{-1}\left\{E\left(f\right)\right\}\approx \sum_{i=1}^{n_{s}}I_{i}\cdot p_{\alpha_{i}}\left(r-r_{i}\right),$$

where $p_{\alpha_{i}}\left(r\right)$ is a waveform function whose exact form depends on $\alpha_{i}$ and the radar parameters (e.g., sinc-like for the specular reflection case $\alpha_{i}=0$).

It is worth noting that the exact GTD-derived waveform is not perfectly represented by Gaussian kernels. Actual scattering responses exhibit oscillatory structures and side lobes, and their exact forms depend on target geometry, aspect angle, and radar parameters. However, in this work, we are not attempting to perform rigorous electromagnetic inversion. Instead, this study adopts Gaussian basis functions as a differentiable, smooth approximation to capture the dominant energy concentration of each scattering response. This approximation enables end-to-end learning while providing an approximate description of dominant peak locations and intensities.

Accordingly, we model the reconstructed HRRP as a sum of Gaussian functions:

(3) $$\hat{x}\left[r\right]=\sum_{i=1}^{n_{s}}I_{i}\cdot \exp\left(-\frac{{\left(r-P_{i}\right)}^{2}}{2{\hat{\sigma }}^{2}}\right),\;r=1,2,\ldots ,L,$$

where *r* indexes the range bin, $\hat{x}\left[r\right]$ is the reconstructed amplitude at that bin, and $\hat{\sigma }$ is a learnable width parameter, initialized to 0.02, shared across all peaks and samples, and clamped to [0.005,0.1] during training. The estimated parameters $\left\{P_{i},I_{i}\right\}$ are intended to characterize dominant HRRP peak distributions in an approximate and differentiable manner. In this formulation, the peak positions $P_{i}$ are represented as continuous normalized range coordinates. During reconstruction, the Gaussian kernels are evaluated on a fixed discrete range grid uniformly sampled over [0,1]. Each peak therefore contributes smoothly to all HRRP sampling positions through continuous Gaussian responses rather than hard bin assignment operations. No explicit rounding or conversion from continuous coordinates to discrete range-bin indices is performed. Consequently, the reconstruction process remains fully differentiable, allowing gradients to be directly backpropagated through the peak position parameters while alleviating potential grid-mismatch and sub-bin quantization effects.

### 3.4. Feature Fusion Module

After obtaining the feature vectors $F_{d}$ and $F_{p}$, the model fuses them using an attention-based mechanism. The two feature vectors are concatenated and then processed by a multi-head attention layer, which learns the contextual relationships between the two branches. The attention operation is defined as

(4) $$\mathrm{Attention}\left(Q,K,V\right)=\mathrm{softmax}\left(\frac{QK^{⊤}}{\sqrt{d_{k}}}\right)V,$$

where *Q*, *K*, and *V* are derived from the concatenated features. Compared with direct concatenation, the attention mechanism enables adaptive weighting between data-driven representations and peak-based features under different target conditions and noise levels. This interaction allows the network to adaptively balance the contributions of the two branches. The output of the attention layer is passed through a feed-forward network. The resulting weights are applied to $F_{d}$ and $F_{p}$, and the weighted vectors are concatenated. A final fully connected layer produces the classification output.

### 3.5. Multi-Loss Function

The network is trained end-to-end by optimizing a loss function that jointly optimizes recognition performance and reconstruction consistency of the estimated peak parameters. The overall loss consists of three terms: classification loss $L_{class}$, sparsity loss $L_{sparse}$, and reconstruction loss $L_{recon}$.

Classification Loss $L_{class}$: We use the standard cross-entropy loss $L_{CE}$:

(5) $$L_{CE}=-\frac{1}{B}\sum_{i=1}^{B}\log\left(\frac{\exp(z_{i,y_{i}})}{\sum_{c=1}^{C}\exp\left(z_{i,c}\right)}\right),$$

where *B* is the batch size, *C* is the number of classes, $y_{i}$ is the true label, and $z_{i,c}$ is the logit for class *c*. Let $\Phi_{d}$, $\Phi_{p}$, and $\Phi_{f}$ denote the data-driven branch, physics-guided branch, and fusion module, respectively. The physics-guided branch $\Phi_{p}$ takes the estimated peak parameters $s_{i}=\left\{P_{i},I_{i}\right\}$ as input. The classification loss is then defined as $L_{class}=L_{CE}$, where $z_{i,c}={\left[\Phi_{f}\left(\Phi_{d}\left(x_{i}\right),\Phi_{p}\left(s_{i}\right)\right)\right]}_{c}$.

Sparsity Loss $L_{sparse}$: To encourage sparsity in the estimated intensity parameters, we apply an $\ell_{1}$ penalty. For a batch of *B* samples, let $s_{i}\in R^{n_{s}}$ denote the intensity vector $\left\{I_{i}\right\}$ for the *i*-th sample. The sparsity loss is defined as the average $\ell_{1}$-norm over the batch:

(6) $$L_{sparse}=\frac{1}{B}\sum_{i=1}^{B}{∥s_{i}∥}_{1}.$$

This formulation directly promotes a sparse vector by driving the majority of its elements toward zero. This loss promotes sparse intensity distributions by encouraging only a limited number of dominant peak components to remain active. Its primary advantage lies in a dual role: it directs the model’s focus to a limited number of range cells, enhancing feature discriminability, while also acting as a regularizer to improve robustness under noisy conditions.

Reconstruction Loss $L_{recon}$: The reconstruction loss measures the mean squared error between the input HRRP and its reconstruction:

(7) $$L_{recon}=\frac{1}{B}\sum_{i=1}^{B}{∥x_{i}-{\hat{x}}_{i}∥}^{2},$$

where ${\hat{x}}_{i}$ is the reconstructed HRRP from the estimated peak parameters. This loss regularizes the peak parameters by enforcing consistency with the dominant HRRP peak envelope.

Total Loss: The total loss is a weighted sum:

(8) $$L_{all}=L_{class}+\lambda_{1}L_{sparse}+\lambda_{2}L_{recon},$$

where $\lambda_{1}$ and $\lambda_{2}$ are hyperparameters balancing the sparsity and reconstruction regularization terms.

## 4. Experimental Results and Analysis

### 4.1. Experimental Settings

This paper presents an extensive experimental investigation on measured HRRP datasets. Specifically, the dataset consists of HRRP signals from 10 distinct measured aircraft categories, namely, A319, A320, A321, A330-2, A330-3, A350-941, B737-7, B737-8, B747-89L, and CRJ-900.

**Figure 2:**
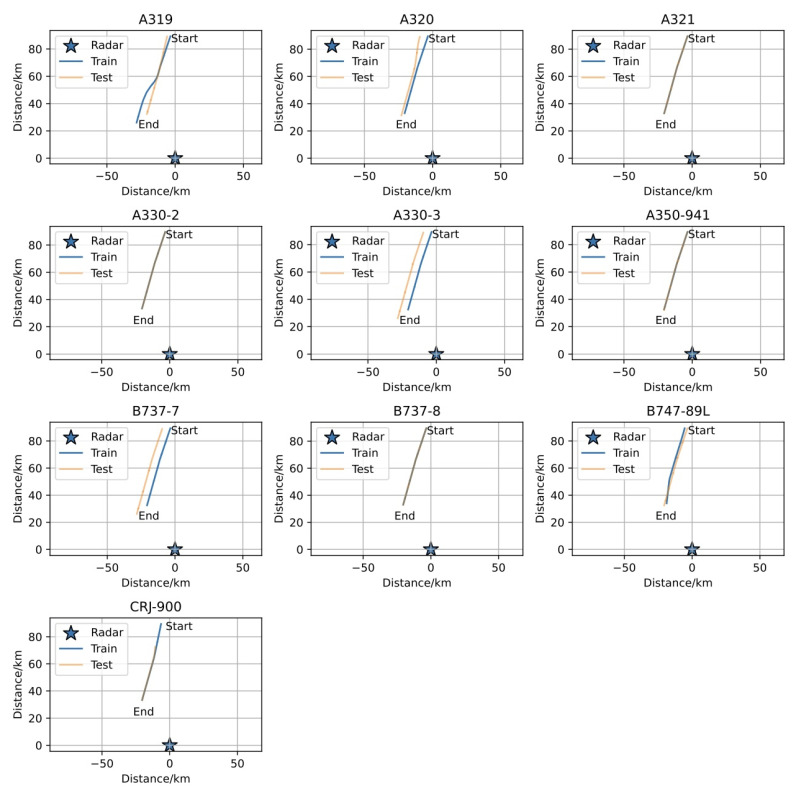
Visualization of data acquisition trajectories for each aircraft model. This figure shows the consistent radar irradiation range (Start-End) and azimuth angle distribution for Train and Test data, demonstrating the fixed aspect angle characteristics of the dataset.

Radar System and Data Acquisition Parameters: All measured HRRP samples are collected from a wideband radar platform. Its main technical specifications are listed below: a working bandwidth of 400 MHz, vertical transmit and vertical receive (VV) polarization, elevation angles between 4.9° and 15.9°, and target azimuth angles distributed within a small sector near 0° (radar north). See Figure 2 for further details. The SNR of the original HRRP samples ranges from 15 to 25 dB, with over 90% of the samples concentrated at 20 dB. These settings are consistent with the actual working state of radar systems for aerial target detection.

Data Distribution and Aspect Angle Consistency: The HRRP data of all 10 aircraft categories were collected from different flight batches along the same fixed route to ensure consistency in data acquisition conditions. As illustrated in Figure 2, the flight trajectories of different aircraft types are highly overlapped, resulting in nearly identical azimuth angle distributions between the training and test sets. This corresponds to a fixed-route aerial target detection scenario. Therefore, the impact of aspect angle variation is not the primary focus of this study.

Aircraft Dimensions and Data Splitting: The key dimensional parameters of these aircraft models are listed in Table 1. For each category, a balanced data split is adopted with 3000 training samples and 1000 test samples, ensuring sufficient data for model training and reliable evaluation.

To eliminate the inherent translation and amplitude sensitivities of HRRP, all samples undergo two critical preprocessing steps: gravity alignment to eliminate translation variations, and $l_{2}$-normalization to mitigate amplitude discrepancies across samples. The $l_{2}$-normalization is mathematically expressed as

(9) $$x_{\mathrm{norm}}=\frac{x}{{∥x∥}_{2}}.$$

**Figure 3:**
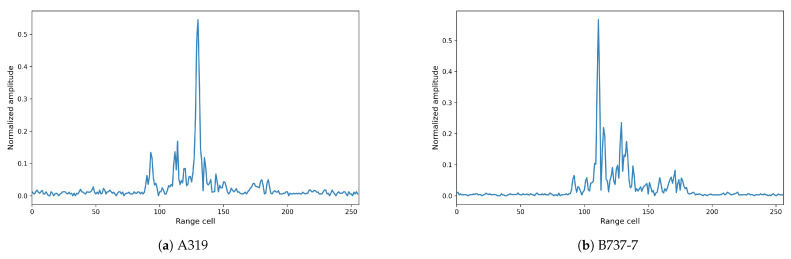
Two HRRP examples used in the experiments (aligned and normalized).

Representative examples of preprocessed HRRP signals from two aircraft categories are visualized in Figure 3.

For the model training and optimization process, the initial learning rate is set to 0.1, which is decayed to one-tenth of its original value every 30 training epochs, with the maximum number of training epochs capped at 100. Additionally, all experiments are implemented using PyTorch (v2.5.1) and conducted on a GeForce RTX 4090 graphics card.

All comparative experiments and ablation experiments are conducted with five independent repeated runs under different random seeds, and the results are reported as the mean value to ensure statistical reliability and reproducibility. The hyperparameters of PGDBF are determined via a grid search to ensure optimal recognition performance: the $\lambda_{1}$ and $\lambda_{2}$ are set to 0.01 and 0.5, respectively, and the batch size is fixed at 128.

### 4.2. Fundamental Experiments

#### 4.2.1. Performance Comparison with Baseline Models

In this subsection, we aim to comprehensively evaluate the 10-class HRRP recognition performance of the proposed method. For performance evaluation, we carry out systematic comparative experiments with multiple state-of-the-art baselines from different research branches, aiming to demonstrate the superiority of the proposed approach for HRRP feature extraction and target classification.

The baseline methods compared in this subsection cover three core methodological paradigms for HRRP recognition: traditional machine learning methods (GMM [3] and SVM [42]), classical deep learning methods (1D CNN [16], TCNN [10], LSTM [43], and ResNet [44]), and self-attention-based models (standard Transformer [45] and PwAET [19]). We further include several advanced models: TARAN [34], a target-aware recurrent attentional network; SCRAM [35], a stacked CNN–Bi-RNN with attention mechanism; and SSPWave [41], a physics-inspired model integrating wavelet transform and signal subspace projection with SNR prior.

Based on the selected baseline models, the overall recognition performance for the 10-class HRRP task is summarized in Table 2. Quantitative analysis indicates that our PGDBF attains both the highest average recognition accuracy and the smallest standard deviation (Std) among all models. It consistently outperforms recent strong baselines, including PwAET and SSPWave. This result demonstrates the effectiveness of the proposed explicit sparse peak parameterization and cross-attention fusion in the context of our HRRP dataset.

**Figure 4:**
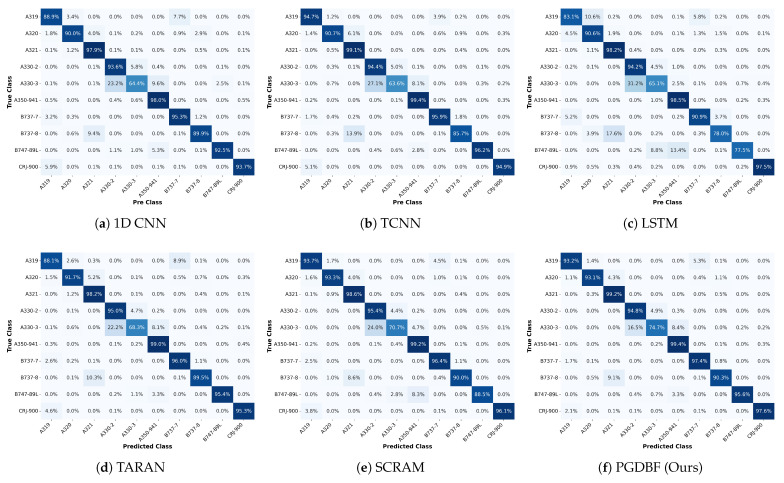
Confusion matrices of various baseline models and the proposed method for HRRP target recognition. The color intensity in each cell indicates the proportion of predictions (darker color: higher proportion; lighter color: lower proportion).

To further unravel the drivers behind this performance edge, we focus on its ability to distinguish targets with highly similar structural configurations—an inherently challenging task in HRRP recognition. Confusion matrices for selected baseline models are visualized in Figure 4. A consistent pattern emerges: targets with highly similar structural characteristics, specifically the A330-2 and A330-3 aircraft, induce high misclassification rates across most baseline methods. For instance, the proportion of samples with the true class of A330-3 being misclassified as A330-2 reaches 31.2% for LSTM, as shown in Figure 4c. In stark contrast, the proposed method reduces the misclassification rate to only 16.5%. This notable improvement can be largely attributed to the physics-guided branch, which captures distinctive peak features of each target. These features include differences in the positions and intensities of dominant HRRP peaks between the A330-2 and A330-3. When these peak-related features are fused with raw HRRP features learned by the data-driven branch, the model achieves better feature discriminability for structurally similar targets, as reflected in the lower misclassification rate.

#### 4.2.2. Visualization of Feature Distribution

**Figure 5:**
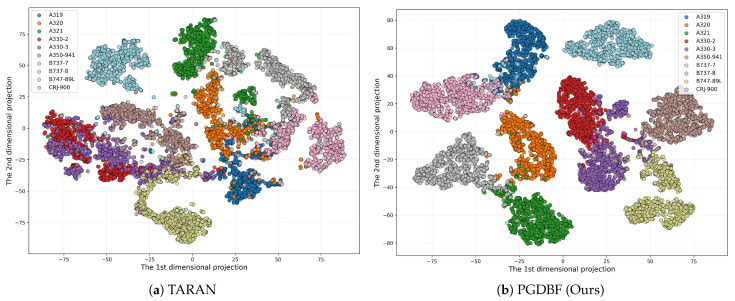
T-SNE visualization of feature space representations for HRRP target recognition.

To further evaluate the quality of feature representations learned by different models, we perform t-distributed Stochastic Neighbor Embedding (t-SNE) on the penultimate layer features of TARAN (a representative data-driven baseline) and the proposed method, projecting the high-dimensional features into a 2D space for intuitive visualization. The results are presented in Figure 5. As shown in Figure 5a, the feature clusters of individual target classes learned by TARAN exhibit noticeable dispersion, with partial overlapping between the features of structurally similar targets. This indicates that the features learned by TARAN lack sufficient discriminability to clearly distinguish between similar targets, which aligns with its relatively high misclassification rate in the confusion matrix analysis. However, the feature embeddings generated by our method in Figure 5b demonstrate well-defined boundaries between different classes: each target category forms a compact and concentrated cluster, and even the features of highly similar targets are distinctly separated. This visualization result further illustrates the superior feature representation capability of the proposed method, which benefits from the fusion of raw HRRP features and peak-related features—endowing the learned representations with stronger intra-class compactness and inter-class separability.

### 4.3. Noise Robustness Experiments

To evaluate robustness against additive Gaussian noise, we conduct recognition experiments under SNR levels ranging from 0 dB to 20 dB. The training set remains noise-free, while the test set is contaminated with different intensities of Gaussian noise. The noise is added to the HRRP samples before $l_{2}$-normalization. The SNR is defined as the ratio of signal power to noise power [46]:

(10) $$\mathrm{SNR}=10\log_{10}\left(\frac{\sum_{i=1}^{D}P_{x_{i}^{\prime }}}{D\times \sigma_{n}^{2}}\right),$$

where $P_{x_{i}^{\prime }}$ denotes the power of the *i*-th sample in the signal, *D* is the total length of the HRRP sequence, and $\sigma_{n}^{2}$ represents the variance of the additive Gaussian noise.

**Figure 6:**
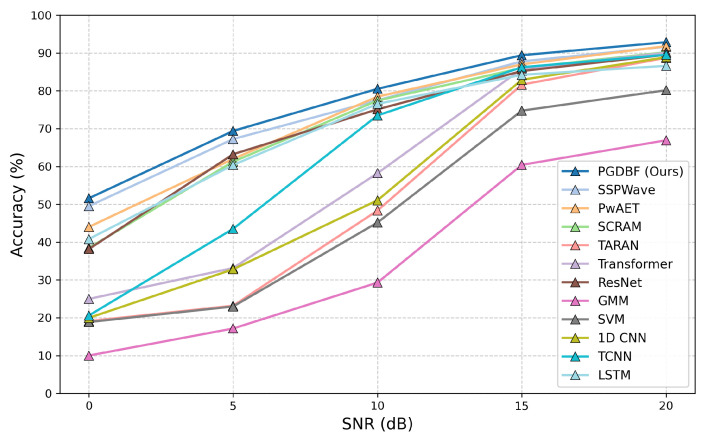
Recognition accuracy of different methods under varying SNR conditions.

As shown in Figure 6, the recognition accuracy of all models increases with SNR. Traditional data-driven methods exhibit significant performance degradation under low-SNR conditions due to the corruption of discriminative HRRP peak distributions by additive Gaussian noise. Compared with recent low-SNR-oriented methods such as PwAET and SSPWave, PGDBF consistently achieves higher recognition accuracy across all evaluated SNR levels, with more pronounced advantages in challenging low-SNR scenarios.

The superior low-SNR performance of PGDBF mainly benefits from the physics-guided peak modeling and the associated reconstruction and sparsity constraints, which help the network focus on dominant HRRP peak responses under noise perturbations. Detailed low-SNR ablation results are further provided in Table 3.

### 4.4. Ablation Experiments

To quantitatively verify the effectiveness of each core component in the proposed PGDBF, we perform a strictly controlled ablation study using the single-variable principle. The recognition results of all configurations are summarized in Table 3. The notations in the table are defined as follows: Data denotes the data-driven feature extraction branch, Physics denotes the physics-guided branch for peak parameter estimation, Fusion indicates the cross-attention feature fusion module, Sparse refers to the sparsity loss constraint, and Recon stands for the reconstruction loss constraint.

The complete PGDBF achieves the highest recognition accuracy under both clean and noisy conditions, verifying the effectiveness of the overall architecture.

Under the noise-free condition, removing either branch leads to a noticeable performance degradation. The single data-driven branch achieves 91.73% accuracy, while the individual physics-guided branch achieves 89.94%. Combining the two branches without cross-attention already improves the accuracy to 92.55%, demonstrating that the physics-guided branch provides complementary structural information beyond purely data-driven features. Furthermore, replacing cross-attention with direct feature concatenation reduces the accuracy from 93.59% to 92.55%, indicating that the proposed cross-attention mechanism can more effectively fuse data-driven representations and physics-guided peak features.

The results under different SNR conditions further reveal the source of the model’s robustness. The data-driven branch suffers severe performance degradation under low-SNR conditions, achieving only 19.95% accuracy at 0 dB. In contrast, the physics-guided branch maintains significantly higher accuracy (43.21% at 0 dB), indicating that sparse peak modeling helps preserve dominant HRRP peak structures under noise perturbations.

However, compared with the complete PGDBF, the individual physics-guided branch still exhibits a noticeable performance gap under low-SNR conditions. This suggests that sparse peak modeling alone mainly preserves dominant structural responses, while some fine-grained discriminative details contained in the original HRRP waveform may be lost. By combining the physics-guided branch with the data-driven branch, PGDBF can simultaneously utilize stable peak structures and complementary detailed features, thereby achieving better low-SNR recognition performance.

The reconstruction and sparsity constraints also play important roles in improving low-SNR performance. Removing the sparsity loss reduces the accuracy from 51.58% to 44.50% at 0 dB, while removing the reconstruction loss decreases the accuracy to 43.56%. These results suggest that the sparsity constraint helps suppress noise-sensitive responses, whereas the reconstruction constraint encourages the estimated peak parameters to preserve the dominant HRRP peak structure.

Compared with these components, removing the cross-attention module causes only a relatively small performance drop under low-SNR conditions, indicating that the primary robustness improvement mainly originates from the physics-guided branch and its associated reconstruction and sparsity constraints.

### 4.5. Hyperparameter Sensitivity Analysis

#### 4.5.1. Sensitivity Analysis of the Number of Peak Parameters $n_{s}$

**Figure 7:**
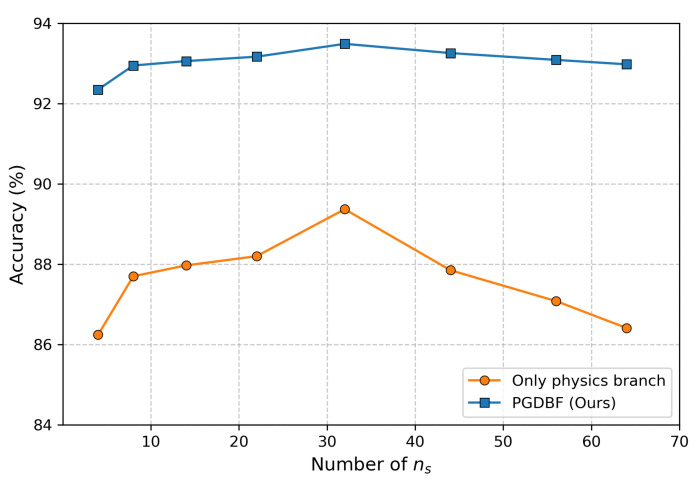
Sensitivity analysis of the preset peak parameter number $n_{s}$ on recognition accuracy.

As a key hyperparameter of the proposed model, the number of preset peak parameters $n_{s}$ defines the modeling capacity of the physics-guided branch. To clarify its selection rationale and the model’s sensitivity to this parameter, we conducted a systematic sensitivity analysis by varying $n_{s}$ over the range {4,8,14,22,32,44,56,64} and evaluating the recognition performance on the measured HRRP dataset. The results are shown in Figure 7, which compares the performance of the physics-guided branch alone and the full dual-branch PGDBF. The optimal value of $n_{s}$ is determined as 32 via grid search, where both branches achieve their highest recognition accuracy.

As observed in the figure, the physics-guided branch alone exhibits a notable performance fluctuation with varying $n_{s}$ (accuracy variation of 3.1%), which aligns with the inherent characteristic of HRRP peak distributions. Generally speaking, the effective number of dominant peaks varies across different targets, and mismatches between the fixed $n_{s}$ and the actual peak characteristics of individual targets will degrade feature representation. In contrast, the proposed PGDBF maintains a highly stable performance across all tested $n_{s}$ values, with an accuracy fluctuation of only 1.1%. This stability is attributed to the complementary effect of the data-driven branch, which effectively mitigates the physics-guided branch’s sensitivity to $n_{s}$ by learning data-specific patterns. Specifically, the dual-branch architecture allows the model to adaptively activate only the meaningful peak components for each target and suppress redundant ones, even with a fixed $n_{s}$. These results demonstrate that PGDBF is robust to moderate variations in $n_{s}$.

A small $n_{s}$ restricts the physics-guided branch’s ability to capture the peak distribution characteristics of complex targets. An overlarge $n_{s}$ brings redundant modeling complexity but no performance improvement. The selected value $n_{s}=32$ balances the modeling capacity and computational efficiency of the physics-guided branch. Therefore, $n_{s}=32$ is a reasonable and reliable hyperparameter for all targets.

#### 4.5.2. Sensitivity Analysis of Loss Weights

**Figure 8:**
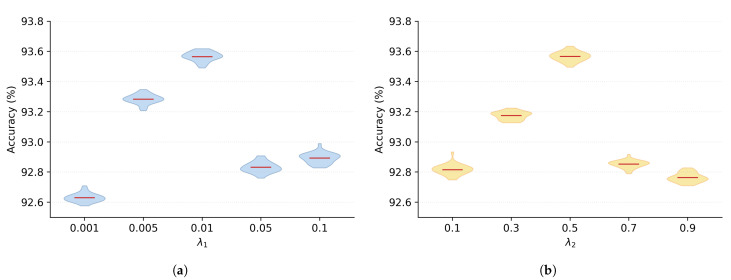
Sensitivity analysis of loss weights on recognition accuracy in the noise-free scenario. (**a**) Sensitivity of $\lambda_{1}$; (**b**) sensitivity of $\lambda_{2}$. The violin plots show the accuracy distribution of 5 repeated experiments, and the marked points represent the mean recognition accuracy.

To clarify the selection rationale and robustness of the loss weights $\lambda_{1}$ (sparse loss) and $\lambda_{2}$ (reconstruction loss), we conduct sensitivity experiments via the control variable method on the noise-free measured dataset. For $\lambda_{1}$, we fix $\lambda_{2}=0.5$ and vary $\lambda_{1}$ in the range of 0.001, 0.005, 0.01, 0.05, 0.1. For $\lambda_{2}$, we fix $\lambda_{1}=0.01$ and vary $\lambda_{2}$ in the range of 0.1, 0.3, 0.5, 0.7, 0.9. All experiments are repeated five times with different random seeds to ensure statistical reliability, and the experimental results reflecting the accuracy distribution are illustrated by violin plots in Figure 8.

For $\lambda_{1}$, the proposed PGDBF achieves the highest and most stable accuracy at $\lambda_{1}=0.01$. A small $\lambda_{1}$ fails to enforce sufficient sparsity of the estimated peak intensities. An overlarge $\lambda_{1}$ suppresses valid peak features and reduces model performance. For $\lambda_{2}$, the model performs optimally at $\lambda_{2}=0.5$. A small $\lambda_{2}$ weakens the reconstruction consistency constraint. An overlarge $\lambda_{2}$ shifts the optimization focus away from classification. These results confirm that the selected values $\lambda_{1}=0.01$ and $\lambda_{2}=0.5$ ensure both high recognition accuracy and model robustness. Thus, we fix these two parameters in all experiments.

### 4.6. Further Discussion

#### 4.6.1. HRRP Reconstruction and Learned Peak Interpretation

**Figure 9:**
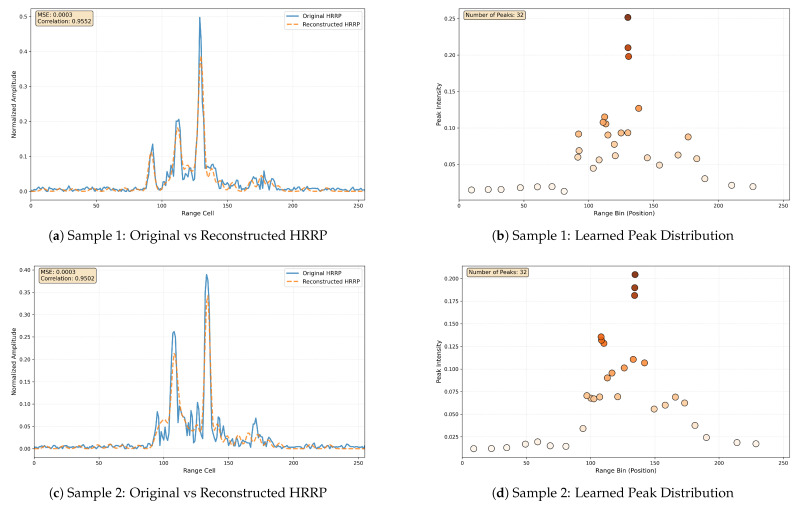
Examples of aligned and normalized HRRP signals for two representative aircraft categories. In (**b**,**d**), different colors of dots indicate the intensity magnitude (lighter colors: lower intensity; darker colors: higher intensity).

To further analyze the interpretability of the proposed PGDBF, Figure 9 presents the reconstructed HRRP waveforms and the corresponding learned peak distributions for two representative samples. Two quantitative metrics are employed to evaluate the reconstruction quality: the reconstruction correlation coefficient, which measures the structural consistency between the reconstructed and original HRRP signals, and the mean squared error (MSE), which evaluates amplitude fidelity.

As illustrated in Figure 9, the reconstructed waveforms exhibit high similarity to the original HRRP signals, particularly in the dominant peak regions. This observation indicates that the learned peak parameters are able to reflect the principal energy distribution characteristics of HRRP signals rather than merely fitting global waveform trends. Since dominant peaks in HRRP are closely related to the major scattering responses of radar targets, the reconstruction results suggest that the proposed peak parameterization mechanism captures representative information associated with dominant scattering-response energy distributions in HRRP. This is conceptually consistent with the physical understanding that dominant scattering centers determine the peak structure of HRRP.

The statistical results over all 10 aircraft categories are summarized in Table 4. All categories achieve reconstruction correlation coefficients above 90.83%, while the MSE values remain below $3.75\times 10^{-4}$. These results demonstrate that the proposed physics-guided branch can consistently characterize dominant HRRP structures across different target categories. More importantly, the learned peak parameters explicitly preserve positional and intensity information, and their distinguishable structural patterns for visually similar targets provide complementary discriminative cues beyond conventional abstract deep features.

Furthermore, the sparsity constraint plays an important role in enhancing representation interpretability. Instead of encouraging the model to fit distributed weak fluctuations or noise-sensitive details, the sparsity regularization biases the network toward a limited number of dominant peak responses. As a result, the learned representations exhibit clearer structural characteristics and improved robustness under noisy conditions, which is also consistent with the superior low-SNR recognition performance observed in previous experiments.

It should be emphasized that the reconstruction module is introduced primarily as an auxiliary consistency constraint rather than a strict electromagnetic scattering inversion model. The core objective of the physics-guided branch is to explicitly estimate sparse peak parameters, including peak positions and intensities, and integrate them into the classification process through cross-attention fusion. Therefore, the proposed framework provides structurally interpretable representations conceptually aligned with electromagnetic scattering principles while preserving the representation capability of deep neural networks.

**Figure 10:**
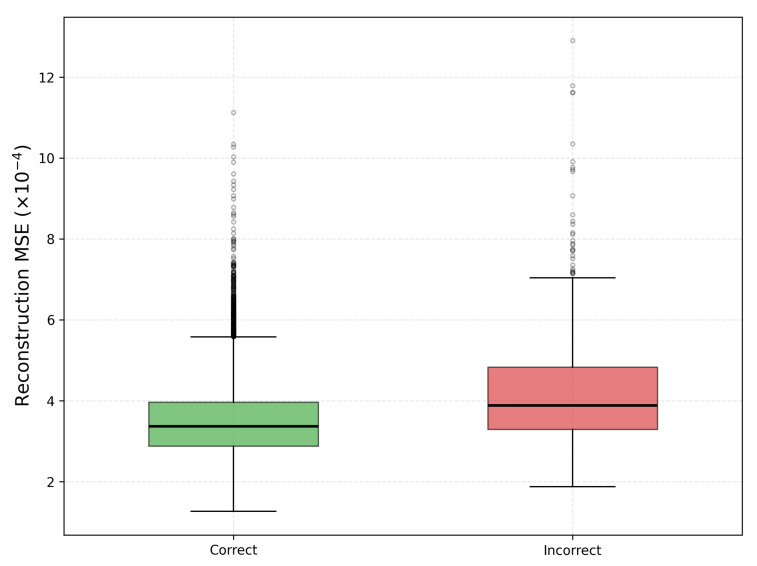
Reconstruction MSE distributions of correctly and incorrectly classified samples.

To further investigate the relationship between reconstruction consistency and classification performance, we compared the reconstruction MSEs of correctly and incorrectly classified samples on the test set. As shown in Figure 10, correctly classified samples exhibit lower reconstruction errors (mean MSE = $3.49\times 10^{-4}$) than misclassified samples (mean MSE = $4.15\times 10^{-4}$), and the difference is statistically significant (p<0.001).

This result indicates that samples with better reconstruction consistency are more likely to preserve discriminative HRRP structures beneficial for classification, further supporting the effectiveness of the proposed reconstruction constraint in learning meaningful peak representations.

Overall, these observations indicate that the proposed PGDBF learns structured peak representations associated with dominant HRRP energy responses rather than purely abstract embeddings. This dual-branch mechanism enables the model to simultaneously achieve strong recognition performance, enhanced robustness, and improved interpretability.

#### 4.6.2. Computational Complexity Analysis

To verify the engineering practicability of the proposed PGDBF, we analyzed the computational complexity of the proposed method and the compared methods using three metrics: floating point operations (FLOPs) representing the computational load, parameters (Params) representing the number of model parameters, and inference time per single sample. The experimental results are shown in Table 5. As observed, the proposed PGDBF introduces a dual-branch structure and cross-attention mechanism, resulting in higher computational complexity than lightweight models such as 1D CNN and TARAN. Nevertheless, compared with the deep sequential architecture of SCRAM, PGDBF achieves moderately lower inference latency despite its more complex dual-branch design. In addition, compared with the Transformer baseline, PGDBF achieves higher recognition accuracy with moderate additional computational cost, demonstrating a favorable trade-off between performance and efficiency. It is worth noting that the proposed PGDBF maintains millisecond-level inference latency (3.16 ms per sample in the current implementation), indicating its potential applicability to practical radar processing scenarios with moderate real-time requirements. Although lightweight models remain faster, the experimental results suggest that the additional computational overhead introduced by the physics-guided branch is still controllable relative to the obtained robustness and interpretability improvements.

## 5. Conclusions

Current data-driven solutions for HRRP target recognition are restricted by weak interpretability and degraded accuracy in noisy conditions. This paper proposed the PGDBF model to mitigate the above drawbacks. By fusing data-driven feature extraction and physics-aware peak parameter estimation and employing cross-attention to achieve adaptive feature aggregation, PGDBF builds a connection between deep learning and radar scattering priors. Experimental results on measured HRRP data demonstrate that PGDBF achieves higher recognition accuracy and improved representation interpretability compared to traditional methods, validating the effectiveness of differentiable peak parameter estimation with reconstruction and sparsity constraints.

Furthermore, PGDBF maintains stable recognition performance across a wide SNR range under additive Gaussian noise perturbations. This improved stability is mainly attributed to the physics-guided branch, which explicitly models sparse dominant peak structures in HRRP signals. By encouraging the network to focus on structurally significant peak components rather than unstable noise-induced responses, the proposed framework captures more reliable target characteristics and improves robustness to low-SNR perturbations.

Despite these contributions, several limitations should be acknowledged. All experiments are conducted on a single measured dataset with highly overlapping azimuth angle distributions between training and test sets, corresponding to a fixed-route scenario. In addition, although the proposed framework maintains millisecond-level inference latency, further validation under broader acquisition conditions and more realistic operational environments is still necessary before practical deployment. Future work will therefore investigate the generalization capability of PGDBF under larger aspect-angle variations and more complex radar interference conditions.

## Figures and Tables

**Table 1:** Dimensions (m) of different aircraft models.

Category	Wingspan	Fuselage Length	Fuselage Height
A319	34.09	33.84	11.76
A320	34.10	37.57	11.76
A321	34.10	44.51	11.76
A330-2	60.30	58.80	17.40
A330-3	60.30	63.60	16.85
A350-941	64.75	66.80	17.05
B737-7	35.80	33.60	12.50
B737-8	35.80	39.50	12.50
B747-89L	68.50	76.40	19.30
CRJ-900	23.20	36.19	7.57

**Table 2:** Recognition accuracy comparison of different methods (mean of ±Std).

Method	Accuracy (%)
GMM [3]	67.72 ± 0.72
SVM [42]	82.45 ± 0.68
1D CNN [16]	90.51 ± 0.44
TCNN [10]	91.53 ± 0.41
LSTM [43]	87.32 ± 0.64
ResNet [44]	91.86 ± 0.47
Transformer [45]	92.08 ± 0.36
TARAN [34]	91.39 ± 0.49
SCRAM [35]	92.27 ± 0.37
PwAET [19]	92.51 ± 0.35
SSPWave [41]	92.68 ± 0.30
PGDBF (Ours)	93.59 ± 0.25

**Table 3:** Ablation study of PGDBF components on the noise-free dataset and under different SNR levels. Recognition accuracy (%). ✓ indicates the component is used; × indicates it is not used.

Settings	Data	Physics	Fusion	Sparse	Recon	Acc. (Clean)	SNR (dB)				
							0	5	10	15	20
Only data branch	✓	×	×	×	×	91.73	19.95	32.81	51.05	86.91	90.87
Only physics branch	×	✓	×	✓	✓	89.94	43.21	58.60	74.36	86.55	89.14
w/o $L_{sparse}$	✓	✓	✓	×	✓	91.96	44.50	59.47	75.32	86.25	91.45
w/o $L_{recon}$	✓	✓	✓	✓	×	92.29	43.56	58.12	74.04	85.54	91.87
w/o cross-attention	✓	✓	×	✓	✓	92.55	50.92	68.74	80.28	89.02	92.18
PGDBF (Ours)	✓	✓	✓	✓	✓	93.59	51.58	69.39	80.52	89.41	92.85

**Table 4:** HRRP reconstruction results for 10 aircraft categories (mean of ± Std).

Category	Correlation Coefficient (%)	MSE ($\times 10^{-4}$)
A319	93.92 ± 0.23	3.26 ± 0.15
A320	92.26 ± 0.17	3.07 ± 0.12
A321	93.51 ± 0.26	2.41 ± 0.18
A330-2	91.04 ± 0.19	3.62 ± 0.14
A330-3	90.83 ± 0.28	3.75 ± 0.21
A350-941	93.42 ± 0.15	2.48 ± 0.11
B737-7	92.87 ± 0.21	2.79 ± 0.16
B737-8	91.36 ± 0.18	3.48 ± 0.13
B747-89L	92.54 ± 0.29	2.96 ± 0.22
CRJ-900	93.51 ± 0.13	2.41 ± 0.09

**Table 5:** Computational complexity comparison of different methods.

Method	FLOPs (M)	Params (M)	Time (ms)
1D CNN [16]	0.46	0.14	0.12
TCNN [10]	3.12	0.65	0.35
TARAN [34]	0.27	0.07	0.18
LSTM [43]	6.58	0.53	1.10
Transformer [45]	9.13	0.89	1.75
SCRAM [35]	75.10	1.01	3.51
PGDBF (Ours)	16.81	1.66	3.16

## Data Availability

The data that support the findings of this study are not openly available due to reasons of sensitivity. Further inquiries can be directed to the corresponding author.
